# Transgenerational effects increase the vulnerability of a host–parasitoid system to rising temperatures

**DOI:** 10.1111/1365-2656.70112

**Published:** 2025-08-12

**Authors:** Natalie L. Bright, Jinlin Chen, J. Christopher D. Terry

**Affiliations:** ^1^ Department of Biology University of Oxford Oxford UK; ^2^ School of Life Sciences Nanjing University Nanjing China

**Keywords:** climate change, *Drosophila*, host‐parasitoid, modelling, population dynamics, transgenerational

## Abstract

1. Transgenerational effects, non‐evolutionary processes by which environmental conditions in one generation influence the performance in subsequent generations, are hypothesised to have substantial consequences for population dynamics under stochastic environments. However, any direct apparent detriment or advantage these processes generate for a focal species may be counteracted by concurrent effects upon interacting species.
2. Using an experimental *Drosophila*–parasitoid model system, we determined how the previous generation's thermal environment impacts the thermal performance of both hosts and parasitoids. We found substantial responses in both trophic levels, with potential evidence for both condition‐transfer effects and adaptive transgenerational plasticity.
3. We used these results to parameterise discrete‐time simulation models to explore how transgenerational effects of thermal conditions and temporal autocorrelation in temperature are expected to impact the time to extinction for this host–parasitoid system under climate change. The models predicted that transgenerational effects would significantly hasten the time to extinction, largely through a reduction in estimated average performance. Under the assumptions of one of the population dynamics models trialled, we identified an additional hastening of extinction from the combined effect of both host and parasitoid transgenerational effects.
4. Our research demonstrates how community‐level consequences of transgenerational effects may impact a population's sensitivity to climate change under a fluctuating environment and highlights the need to quantify and contextualise thermal transgenerational effects in their ecological setting.

## Graphical abstract

Insect thermal performance can be modulated by temperatures experienced by preceding generations, but it is unclear how these effects may scale up to communities. Using an experimental *Drosophila*–parasitoid system and simulations, transgenerational effects affecting both trophic levels are shown to have emergent impacts on persistence under rising temperatures.

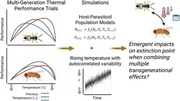

## 1 INTRODUCTION

Transgenerational effects, where the conditions experienced in earlier generations impact the performance in subsequent generations without evolutionary change (Galloway & Etterson, 2007), are increasingly recognised as an influential determinate of climate change risk (Donelson et al., 2018). Where there is autocorrelation in environmental fluctuations facing successive generations (Heino et al., 2000; Petchey et al., 1997), adaptive transgenerational effects may help populations to persist or alternatively could shorten persistence if negative impacts accumulate through condition‐transfer. However, the impact of transgenerational effects in a multispecies context has been underexplored (Malinski et al., 2024) despite wide appreciation that climate change effects will be modulated by the wider community (Carroll et al., 2024; Chen & Lewis, 2023; Gilman et al., 2010; Tylianakis et al., 2008). The impact of transgenerational effects in a focal species response may be offset or exacerbated by corresponding responses of interacting species.

Transgenerational effects can include both condition‐transfer and adaptive plasticity effects through anticipatory effects, which can be challenging to disentangle (Sánchez‐Tójar et al., 2020). Condition‐transfer, the direct modulation of the investment the parental generation can make in the next, is likely the most widespread form of transgenerational effect (Bonduriansky & Crean, 2018). Anticipatory effects, on the other hand, occur when parents are able to actively influence their offspring's phenotypes to suit the anticipated environment for the next generation (Burgess & Marshall, 2014). Such priming could reduce the lag between environmental signal and phenotypic response (Cavieres et al., 2020) when environmental fluctuations are predictable, thus significantly altering the extinction risk posed by climate change (Salinas & Munch, 2012). However, the supporting evidence for the prevalence of transgenerational effects is still relatively weak (Sánchez‐Tójar et al., 2020; Uller et al., 2013) and the magnitude and direction are context‐dependent (Baker et al., 2019; Yin et al., 2019). Furthermore, identifying a transgenerational effect is only the first step to demonstrating impact—the effect must be contextualised into an environmental (Burgess & Marshall, 2014) and biotic context (Walther, 2010).

Here, we investigate the impact of transgenerational effects in a model experimental *Drosophila* host–parasitoid system. Insects are likely to be amongst the most responsive taxa to climate change (Harvey et al., 2023). Parasitoids are ectotherms whose larvae develop by feeding in or on the bodies of other arthropods, representing ~10% of described insect species (Eggleton & Belshaw, 1997; Godfray, 1994). Many of the processes intrinsic to parasitoids' life history demonstrate temperature dependence; for instance, foraging traits, such as successfully locating and ovipositing in a host egg, and attack traits, such as evading the host's immune defences (Hance et al., 2007; Synodinos et al., 2021). Furthermore, various responses to fluctuating temperature have been documented in parasitoid attack rates (Costaz et al., 2023; Ismaeil et al., 2013; Le Lann et al., 2021). The potential impact of transgenerational effects on insect fitness has long been acknowledged (Mousseau & Dingle, 1991) and has been demonstrated in *Drosophila* in a variety of contexts (Cavieres et al., 2020; Huey et al., 1995; Schiffer et al., 2013). By strongly regulating arthropod host population dynamics and coexistence between different host species (Godfray, 1994; Terry et al., 2021), parasitoids play important roles in maintaining biodiversity. Host–parasitoid interactions thus represent an ecologically important subset of biotic interactions, yet are relatively underrepresented in the literature on biotic responses to climate change (Furlong & Zalucki, 2017; Hance et al., 2007; Jeffs & Lewis, 2013; Thierry et al., 2019). Host–parasitoid interactions offer an experimentally amenable window to start to parse ecosystem complexity. Since parasitoid reproduction is closely linked to their success finding and attacking hosts (Hassell, 2000), the dynamics between host and parasitoid populations are tightly coupled with the high potential for emergent effects to arise in response to climate change (Furlong & Zalucki, 2017; Thierry et al., 2021).

The environmental temporal autocorrelation is central to the impact of transgenerational effects on population dynamics. Without autocorrelation, if the environment faced by a particular generation is not at all predictable from the previous generation, transgenerational effects can be well summarised by a long‐term average. One route to isolate the impact of considering transgenerational effects is to identify emergent effects on population dynamics with and without autocorrelation. However, identifying an emergent impact on population dynamics is complicated by the fact that autocorrelation can in itself impact population persistence (Lande et al., 2003; Postuma et al., 2020). Teasing out the impact of explicitly considering transgenerational effects for population dynamics therefore requires the careful comparison of multiple models. To explore the role of transgenerational effects within a tightly coupled trophic system responding to generation‐scale variance in temperature, we first conducted highly replicated experimental trials to assess how the thermal performance curves of both host and parasitoid are influenced by the temperatures the previous generation is exposed to. We test whether including the temperature experienced by the previous generation can improve predictions of reproductive rate. Focussing on the impacts on population persistence under warming conditions, we then parametrised simulation models to explore the impact of the observed transgenerational effects. We hypothesised that there would be an extra emergent dynamic impact of the combined transgenerational effects across trophic levels.

## 2 METHODS

### 2.1 Study organisms

Drosophila (Diptera: Drosophilidae) provide a practical system that has been widely used to study both thermal performance and community responses to climate change (Davis et al., 1998; Hoffmann & Sgrò, 2011; Rezende et al., 2020). In *D. melanogaster*, a significant interaction between parental and offspring thermal environment on the entire thermal performance curve has been previously identified (Cavieres et al., 2019; Gilchrist & Huey, 2001; Huey et al., 1995). Compared to other model systems of phenotypic plasticity (e.g. Fey et al., 2021), our use of *Drosophila* allows for the discretisation of generations and a simplification of the modelling process.

We used *Drosophila sulfurigaster* (Duda, 1923) and a generalist hymenopteran parasitoid wasp *Asobara* (Braconidae: Alysiinae, strain: KHB, reference: USNMENT01557097, BOLD process ID:DROP043‐21, species identifier: drop_Aso_sp8, Lue et al., 2021). *D. sulfurigaster* is a partially susceptible native host of *Asobara* parasitoids, allowing for some variation in the rate of successful parasitism across treatments. The parasitoid *Asobara* attacks the second‐instar larval stage, with a single offspring emerging from each host pupa. Importantly, *Asobara* (henceforth simply ‘wasps’) has approximately the same generation time as *D. sulfurigaster* (henceforth simply ‘flies’) simplifying both experiments and analysis. Prior to the experiment, the wasps had been maintained on highly susceptible *D. melanogaster* larvae.

Both host and parasitoid species were established from adults collected in the Wet Tropics bioregion of northeast Queensland, Australia. There, the *Drosophila* community suffers considerable parasitoid pressure, with wasp DNA found in between a third to a half of pupae (Jeffs et al., 2021). *Drosophila* and parasitoid cultures were originally exported under permit PWS2016‐AU‐002018 granted by the Department of Energy and Environment of the Australian Government. These cultures had been maintained at a constant 24°C at the Biology Centre, Czech Academy of Sciences, before being transferred to the Department of Biology, University of Oxford, and maintained at 25°C on cornflour–sugar–yeast–agar *Drosophila* food medium in a 12:12 h light: dark cycle for the preceding year. No specific ethical approval was required for the experiments.

### 2.2 Effect of temperature on fly reproduction

**FIGURE 1:**
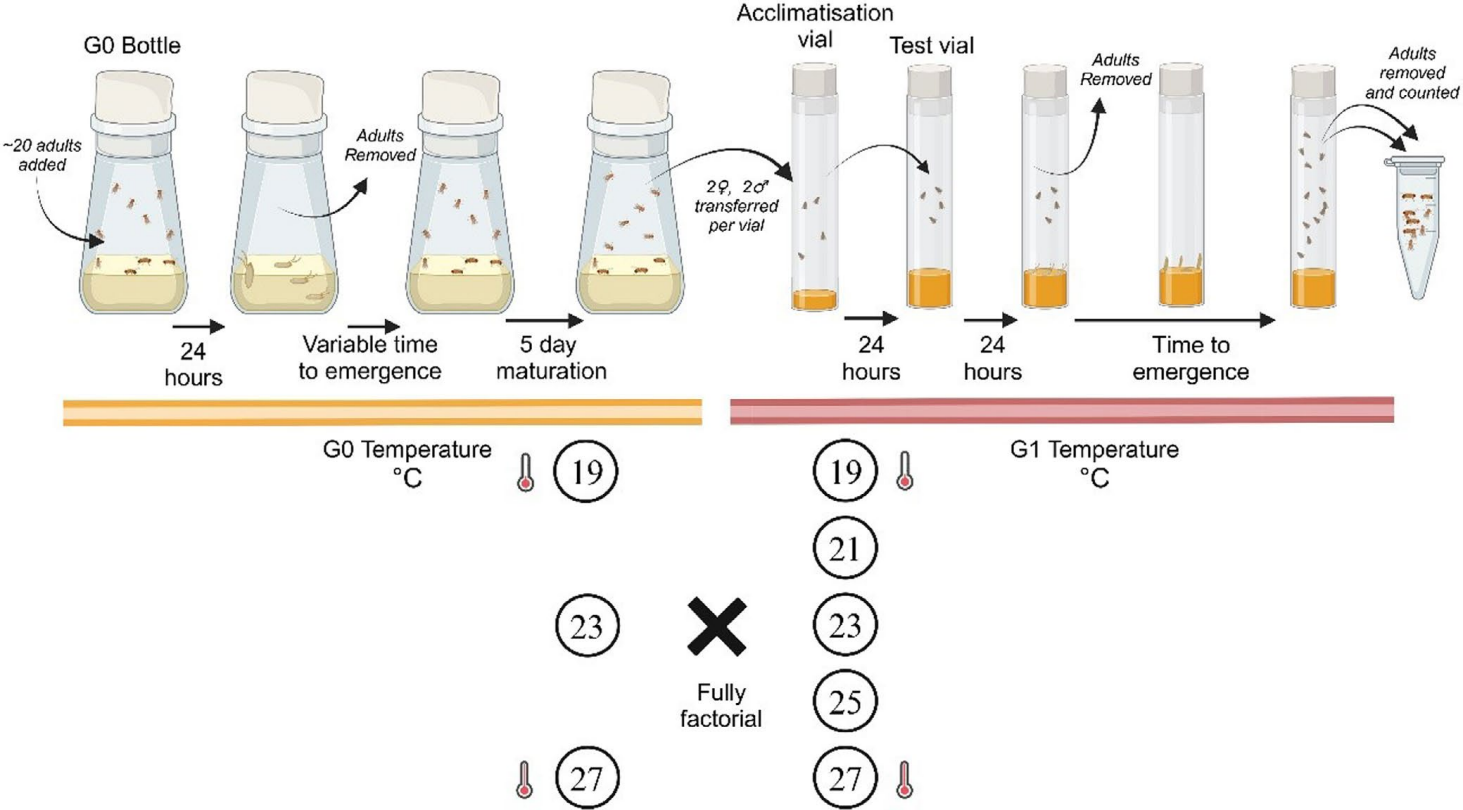
Summary of experimental design for the fly temperature treatment experiment. G0 = Generation 0. G1 = Generation 1. Large numbers of G0 bottles were initiated with approximately 20 flies and randomly assigned to incubators set at one of three G0 temperatures (19, 23 and 27°C). After 24 h to lay eggs, the adult flies were removed. Once G0 flies emerged and matured, two males and two females were counted into each acclimatisation vial. Acclimatisation vials were randomly assigned to incubators set at one of five temperatures (19, 21, 23, 25 and 27°C) in a fully factorial design (*n* = 40 per combination). After 24 h of acclimatisation, flies were transferred into standardised test vials that remained in their assigned G1 test temperature to lay eggs for 24 h. G1 emergences were regularly removed and counted.

The preceding generation of flies (G0) was initiated in 48 bottles, each with 30 mL of food medium. Approximately 20 stock adult flies were allowed to lay eggs for 24 h to generate a low density in each bottle. These bottles were then randomly assigned to one of three incubators (19, 23 and 27°C), under a 12:12 h light: dark cycle (Figure 1). This 8°C range was chosen based on prior knowledge of the community's thermal sensitivity (Chen & Lewis, 2024) to be ecologically relevant and approach the host's reproductive limit. Mean temperatures at the focal area both species originate from range from 21°C at the highest elevation sites to 26°C at the lowest (Jeffs et al., 2021). Temperature and humidity loggers in each incubator showed that a controlled environment was maintained throughout the experiments. Across all the experiments, pupation cards were added to provide a vertical surface for pupae.

Subsequent steps were timed relative to developmental milestones, as time to eclosion is dependent on the temperature treatment (9 days at 27°C, 11 days at 23°C and 15 days at 19°C). Since *D. sulfurigaster* can take 4 days maturation post‐eclosion before laying eggs (Jinlin Chen, *pers. obs*.), 5 days post‐eclosion were allowed in the G0 temperature. Two male and two female G0 flies were counted into acclimatisation vials containing food medium under light CO_2_ anaesthesia (Figure 1). Forty such acclimatisation vials from each G0 temperature were randomly assigned to incubators set at one of five temperatures (offspring generation [G1] temperatures: 19, 21, 23, 25 and 27°C) for 24 h (600 vials total). The acclimatisation vials minimised the impact of the CO_2_ anaesthesia and allowed acclimation to the new temperature.

For the reproduction trials, the flies in each acclimatisation vial were transferred into standardised test vials containing 5 mL of food medium. Any deaths at this stage were noted, but otherwise considered part of the performance assessment. These vials remained at their G1 test temperature. After 24 h of egg laying, the G0 adult flies were removed. Vials were checked regularly, and as G1 flies emerged, they were removed, stored and then counted. In total, 15,755 flies were counted across the 600 trials.

### 2.3 Effect of temperature on wasp reproductive output

**FIGURE 2:**
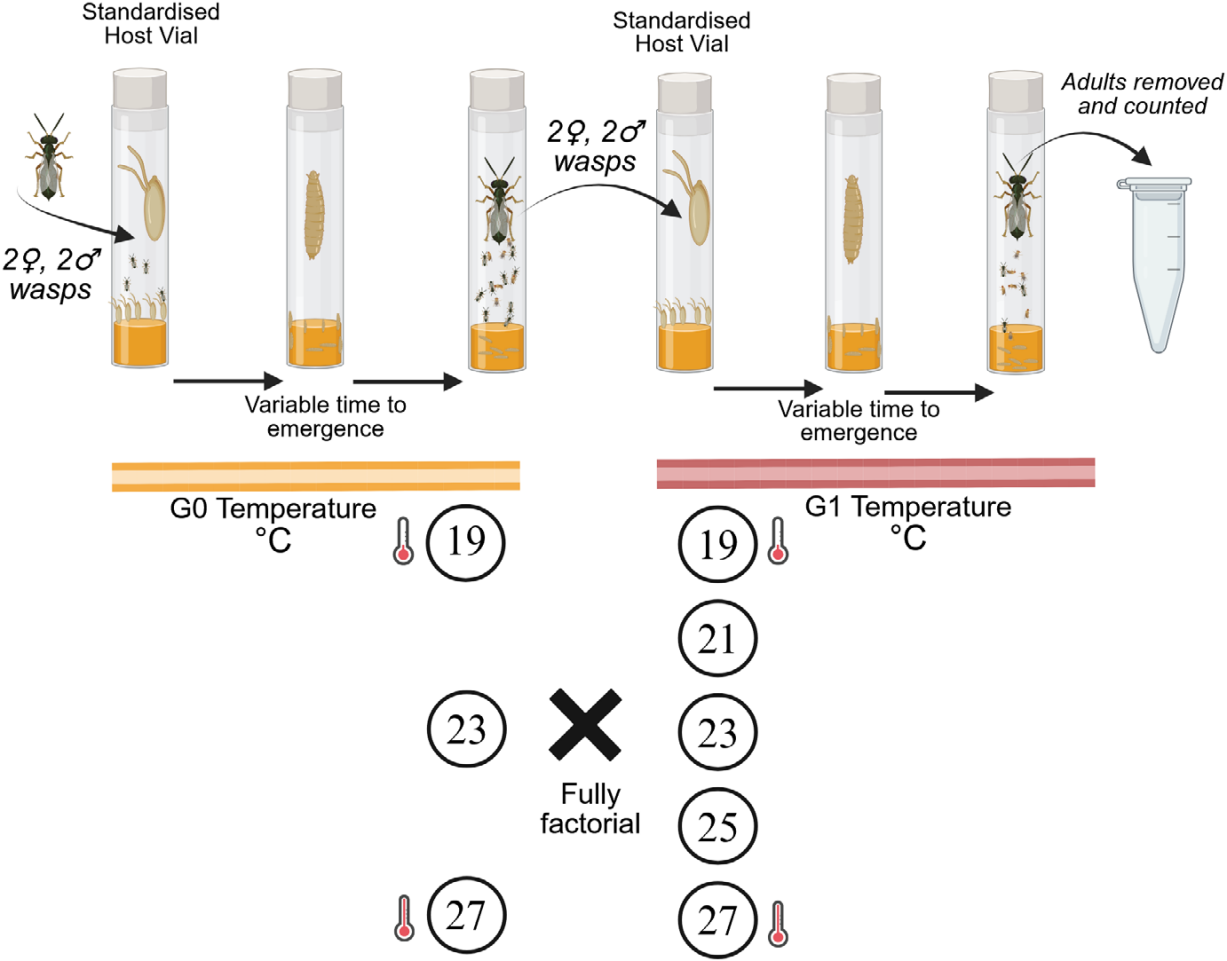
Summary of experimental design for wasp temperature treatment experiment. G0 = Generation 0. G1 = Generation 1. To initiate G0, two male and two female wasps were added to standardised vials of hosts. The vials were stored in incubators at either 19, 23 and 27°C. Two male and two female wasps that recently emerged were transferred to G1 host vials randomly assigned to G1 temperatures (19, 21, 23, 25 and 27°C), in a full factorial design. Each treatment combination had between 16 and 20 replicates. As G1 wasps and flies emerged, they were removed, stored and then counted.

The wasp temperature treatment experiment followed a similar design (Figure 2). Standardised host vials were founded with five adult female flies, sufficient males, 5 mL of standard food medium and a sprinkle of yeast granules to stimulate egg laying and reduce variance in egg laying rate. Vials were maintained at 25°C, and adult flies were allowed to lay eggs for 48 h before being removed. Two male and two female wasps were aspirated into each standardised host vial shortly after initiation to attack the early instar larvae. A total of 48 G0 host vials with wasps were randomly assigned to one of the three G0 temperatures (19, 23 and 27°C, 16 per temperature).

Due to differences in development time, the timing of G1 was staggered. Upon the first observed adult wasp emergence from each G0 treatment, G1 standardised host vials were prepared (as described above), to which two male and two female G0 wasps were added. These vials were randomly assigned to one of the five G1 test temperatures (19, 21, 23, 25 and 27°C), following the fully factorial design. There were sufficient G0 wasps for 16–20 replicates for each treatment combination (totalling 277 vials across all 15 combinations). Test vials were checked regularly and both flies and wasps were removed, stored and counted. In total, 6564 flies and 18,453 wasps were counted.

### 2.4 Statistical analysis

#### 2.4.1 Flies

To test the statistical support for parental generation (G0) temperature influencing fly reproduction rate in the subsequent generation (G1), multiple models were fit and compared by AIC, with and without the inclusion of G0 temperature. A thermal performance curve likelihood function (Equation 1) based on the deutsch_2008 function (Deutsch et al., 2008) in the rTPC package (Padfield et al., 2021) customised to fit to discrete count data,

(1) $$\begin{array}{c} N∼R_{\max}\left(\exp\left(-{\left(\frac{T-T_{\mathrm{opt}}}{2a}\right)}^{2}\right)\right)\text{for}\;T\leq T_{\mathrm{opt}}\\ N∼R_{\max}\left(1-{\left(\frac{T-T_{\mathrm{opt}}}{T_{\mathrm{opt}}-CT_{\max}}\right)}^{2}\right)\text{for}\;T>T_{\mathrm{opt}} \end{array}$$

where N is the number of flies expected and T is the G1 temperature (°C). This function reproduces the expected shape of the thermal performance curve with biologically interpretable parameters. The three fitted parameters are the optimum temperature $T_{\mathrm{opt}}$, the maximum reproduction at optimum temperature $R_{\max}$ and a curve width parameter a. The critical thermal maximum $CT_{\max}$ fell outside the range of the experimental trials and was fixed at 30°C based on previous studies in this species (Chen & Lewis, 2024). To account for the overdispersion in the observations, a negative binomial error function was used.

The model variants were tested where the three fitted parameters ($T_{\mathrm{opt}},\;R_{\max},\;a$) were fixed regardless of the G0 temperature (Model 1b), with the parameters having a linear dependence on G0 temperature (Model 1c) and with the parameters having separate values for each of the three G0 temperature levels (Model 1d). Three further models were tested that included temperature dependence on only two of the three parameters (Models 1e–g).

#### 2.4.2 Wasps

A similar approach was followed to test if G0 temperature influences wasp reproductive output. To account for variation in the numbers of larvae in the standardised host vials, the proportion of wasps of the total emerged insects (wasp count/(wasp count + fly count)) was used, assuming a binomial distribution. This approach assumes that the combined count was independent of G1 temperature, which was supported (linear model of Total Count ~ G1, *F*_1,275_ = 0.373, *p* = 0.541).

Unlike for fly reproduction, there is no established thermal performance model to use for parasitoid wasps. Based on inspection of the data, a monotonic relationship between temperature and reproduction appeared justified within the trial range, and binomial generalised linear models were fit. Multiple models were fit and compared by AIC: without inclusion of G0 temperature (Model 2a), with G0 temperature as a linear predictor (Model 2b) and with an interaction between G0 and G1 temperatures (Model 2c). To examine if the effect of G0 was non‐linear, separate models for each of the three G0 temperatures were fit, and their likelihoods combined (Model 2d).

### 2.5 Simulation modelling

To explore how transgenerational effects of thermal conditions and autocorrelation in temperature may impact the time‐to‐extinction of this host‐parasitoid system under a high‐emissions climate change scenario, we built simulation models incorporating data from the experiments. Both host and parasitoid species are assumed to have discrete and synchronised generations in a Nicholson–Bailey framework (Hassell, 2000).

Separate simulation models were built parameterised either with the model fits with or without including transgenerational effects in either species' performance. Two modelling frameworks were used to explore the potential impact on dynamics: the first (Model A) using a simple Type II functional response, and the second (Model B) using a somewhat more realistic representation of spatial aggregation.

#### 2.5.1 Model A—Type II functional response

The potential fly population in the next time step $N_{t+1}^{*}$, given the current fly population density $N_{t}$ is based on a logistic growth model:

(2) $$N_{t+1}^{*}=\mathrm{\gamma r}\left(T\right)N_{t}\left(1-\frac{N_{t}}{K_{H}}\right)$$

where potential fly population growth rate in a particular environment $r\left(T\right)$ is scaled by a failure rate γ, and $K_{H}$ is a host carrying capacity term.

The number of flies lost to parasitism followed a Type II functional response:

(3) $$N_{t+1}=N_{t+1}^{*}-P_{t}\frac{\alpha \left(T\right)N_{t+1}^{*}}{1+\alpha \left(T\right)hN_{t+1}^{*}}$$

where h is a handling time, and $\alpha \left(T\right)$ is the temperature dependent attack rate.

The wasp population in the next generation was calculated similarly, with the addition of a small immigration component (d) to maintain some wasp threat throughout:

(4) $$P_{t+1}=P_{t}\frac{\alpha \left(T\right)N_{t+1}^{*}}{1+\alpha \left(T\right)hN_{t+1}^{*}}+d.$$

#### 2.5.2 Model B—Proportion surviving under spatial aggregation

An alternative model followed the overall structure of Model A, but models the conversion of potential hosts as a proportion that survive assuming aggregated parasitoid attacks determined by the parameter k, following Hassell (2000) and Meisner et al. (2014):

(5) $$N_{t+1}=N_{t+1}^{*}{\left[1+\frac{\alpha \left(T\right)P_{t}}{k\left(1+\alpha \left(T\right)hN_{t+1}^{*}\right)}\right]}^{-k}.$$

Taking the wasp population as those that do not survive, the wasp population size in the next generation is then:

(6) $$P_{t+1}=N_{t+1}^{*}\left(1-{\left[1+\frac{\alpha \left(T\right)P_{t}}{k\left(1+\alpha \left(T\right)hN_{t+1}^{*}\right)}\right]}^{-k}\right)+d.$$

### 2.6 Experimentally derived temperature dependence

The fly population growth rate $r\left(T\right)$ followed the environmental performance curve fit to the empirical data (Equation 1). Without the inclusion of transgenerational effects, the values of the thermal performance curve parameters were fixed to those identified with Model 1b (Table S3). When transgenerational effects are included, linear interpolation is used between and beyond the curve parameter values identified by the best fit model (Model 1d, Figure S1). To prevent negative *R*_max_ values, the expression was lower‐bounded at 0.1.

To represent temperature dependence in the attack rate $\alpha \left(T\right)$ of the wasps, the proportion models fit to the experimental data $\left(\eta ,\left(T\right)\right)$ were scaled by a maximum wasp attack coefficient *t*_*p*_:

(7) $$\alpha \left(T\right)=\eta \left(T\right)t_{p}$$

Model 2a (which did not include G0 temperature as a predictor) was used for *η*(*T*) without the inclusion of transgenerational effects. To include transgenerational effects, a model equivalent to Model 2d was used, which interpolated between the fitted values of the parameters at the three G0 with a quadratic function.

### 2.7 Simulation model parameter calibration

For both models, remaining parameters were selected based on a combination of experimentally derived values, previous literature and manual tuning to generate plausible dynamics prior to the introduction of simulated climate change (Table S3). Fly carrying capacity *K*_*n*_ was set sufficiently large to overcome demographic stochasticity, whilst fly failure rate γ was set on the same order of magnitude as *R*_max_ to stabilise the overall growth rate and prevent overly explosive exponential growth. Wasp handling time *h* was set at 0.01 as, on average, the wasps can parasitise a maximum of approximately 100 flies due to egg limitation (maximum attack rate is 1/*h*) (Godfray, 1994; Hassell, 2000). The wasp attack coefficient *t*_*p*_ is a scaling factor that defines wasp attack rate and was tuned to generate an initial rate of parasitism of approximately 50% in line with field studies of this system (Jeffs et al., 2021). The wasp spatial aggregation term *k* was set last, with simulations run at a range of values around that in Meisner et al. (2014) to generate plausible dynamics with the populations varying moderately around their carrying capacities.

### 2.8 Generating simulation temperature regime

Simulated temperature trajectories were built on a 500 generation burn‐in period with mean temperature 24°C (to settle any initial transient dynamics), followed by a linear rise of 5°C warming over 2000 generations (approximately 80 years). On top of this, Gaussian noise (σ = 1) was superimposed, with an autocorrelation of either 0 or +0.8 (Figure 5). To prevent numerical errors outside the bounds of the extrapolation, temperatures were capped at 30°C, the flies thermal maximum. These values were selected to align with the ranges and coefficient of variation observed in summer fortnightly averages at a long‐term meteorological station near the species collection site (Koombooloomba Dam). The extended temperature ramp minimises lag‐effects and therefore also allows our results to be approximately interpreted as the mean temperature at which the community is able to persist.

### 2.9 Simulation trials

The simulation models were used to investigate host time‐to‐extinction under a suite of model assumptions: the inclusion of transgenerational effects in each of the flies and wasps, and the inclusion of positive autocorrelation in temperature between generations. Each model scenario was run 100 times with consistent sets of temperature trajectories used across scenarios. Extinction was defined as the first generation in which the fly population fell below one.

We tested for an interaction between the impact of transgenerational effects (in the flies and wasps) and autocorrelation in temperature on (logged) time‐to‐extinction using linear mixed‐effects models. By taking the logarithm, we modelled the effect of the treatments on the proportional change in time‐to‐extinction, thus assuming a multiplicative relationship between variables. The full model included, as fixed effects, autocorrelation (0 or 0.8) and the inclusion of transgenerational effects for each species (ON/OFF), along with all interaction terms. Temperature trajectory seed ID was included as a random effect. The significance of the interaction terms was tested with a Type III ANOVA with Satterthwaite's method via the *lmerTest* package (Kuznetsova et al., 2017).

## 3 RESULTS

### 3.1 Experimental evidence

**FIGURE 3:**
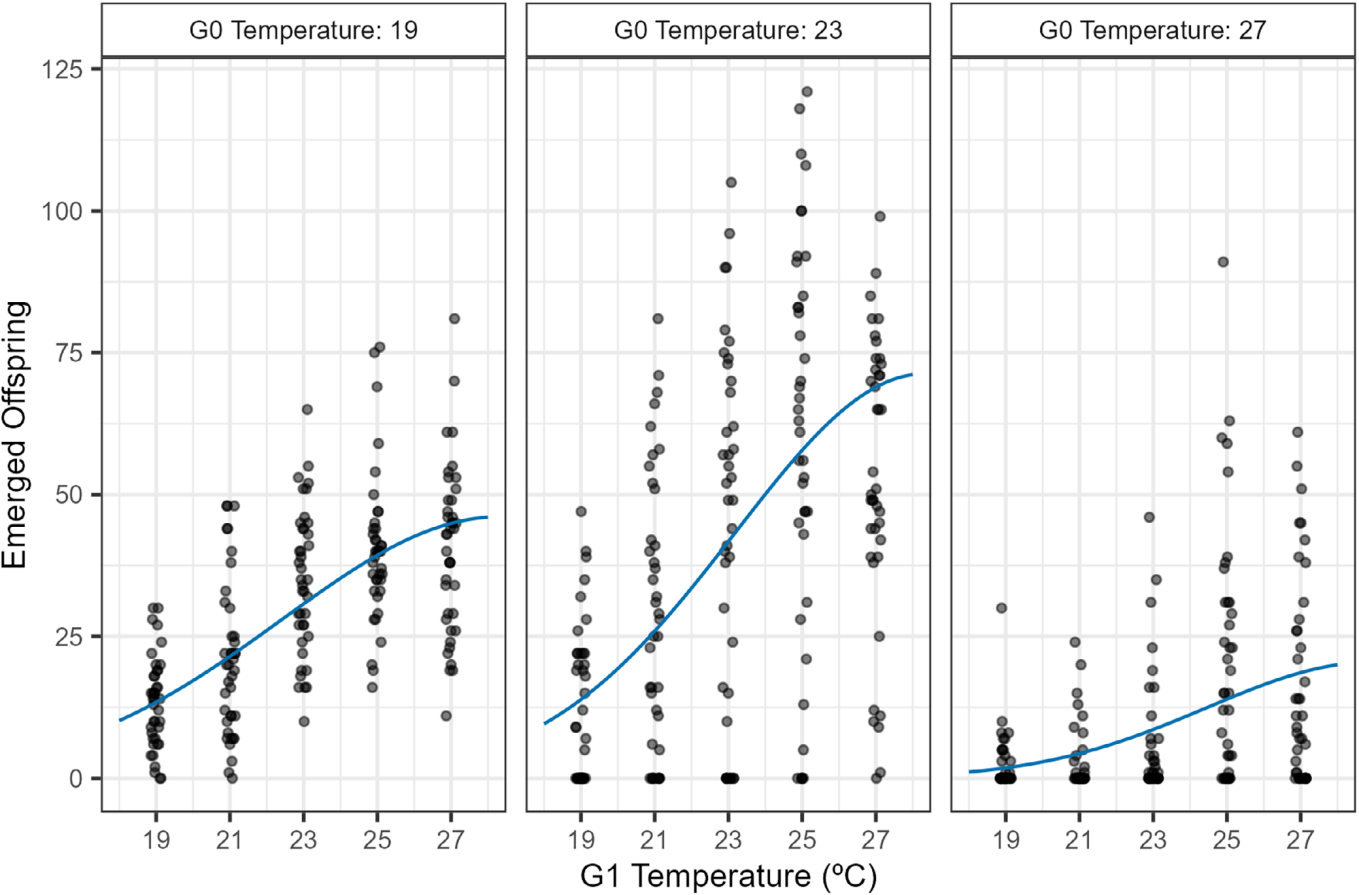
Fly reproduction best fit thermal performance curves and raw data. Each panel shows the effect of temperature during that generation (G1) on the number of emerged offspring, for each temperature in the preceding generation (G0). See (Figure 1) for how generations were delineated. Black points represent raw data from each vial and are jittered horizontally. Means and standard errors for each treatment combination are given in Figure S3. Blue lines represent central expectations based on the best fit thermal performance curve model. Parameter estimates for the thermal performance curves and 95% confidence intervals are in Supporting Information (Table S1).

For the fly temperature treatment experiment, the best model fit included G0 temperature as an explanatory variable, with all parameters able to vary with the three levels of G0 (Table 1, Model 1d), indicating a significant impact of transgenerational effects of thermal conditions on fly performance. As G1 temperature increased, emerged offspring numbers increased for all three fly G0 but at distinct rates (Figure 3). At a G1 temperature of 19°C, the greatest predicted reproduction was seen when G0 = 19°C. At all temperatures above 19°C, the greatest predicted reproduction was seen when G0 = 23°C. When G0 = 27°C, performance was low across the temperature range, suggesting the presence of a non‐adaptive condition‐transfer effect.

**TABLE 1:** Tests of impact of G0 temperature on fly reproduction rates through model comparison.

Model	Error distribution	G0 temperature dependence of parameters			*N* parameters	Log‐likelihood	ΔAIC
		*T*_opt_	*R*_max_	*a*			
1a	Poisson	None			3	−7737.23	10700.77
1b	Negative Binomial	None			4	−2502.36	233.03
1c	Negative Binomial	Linear dependence on G0 Temperature			7	−2429.22	92.76
1d	Negative Binomial	Separately fit for each G0 Temperature			10	−2379.85	—
1e	Negative Binomial	None	Separately fit for each G0	Separately fit for each G0	8	−2427.30	90.91
1f	Negative Binomial	Separately fit for each G0	None	Separately fit for each G0	8	−2482.53	201.36
1g	Negative Binomial	Separately fit for each G0	Separately fit for each G0	None	8	−2440.89	118.10

*Note*: The best supported model (1d) included full non‐linear dependence of the three fitted thermal performance curve parameters on the temperature in the previous generation (G0). Models that included the G0‐temperature dependence for only two of the three parameters (Models e–g), or included a linear G0 temperature dependence (1e), gave notably less good fits (ΔAIC > 90). Using a simpler Poisson error distribution (1a) was not supported at all.

**FIGURE 4:**
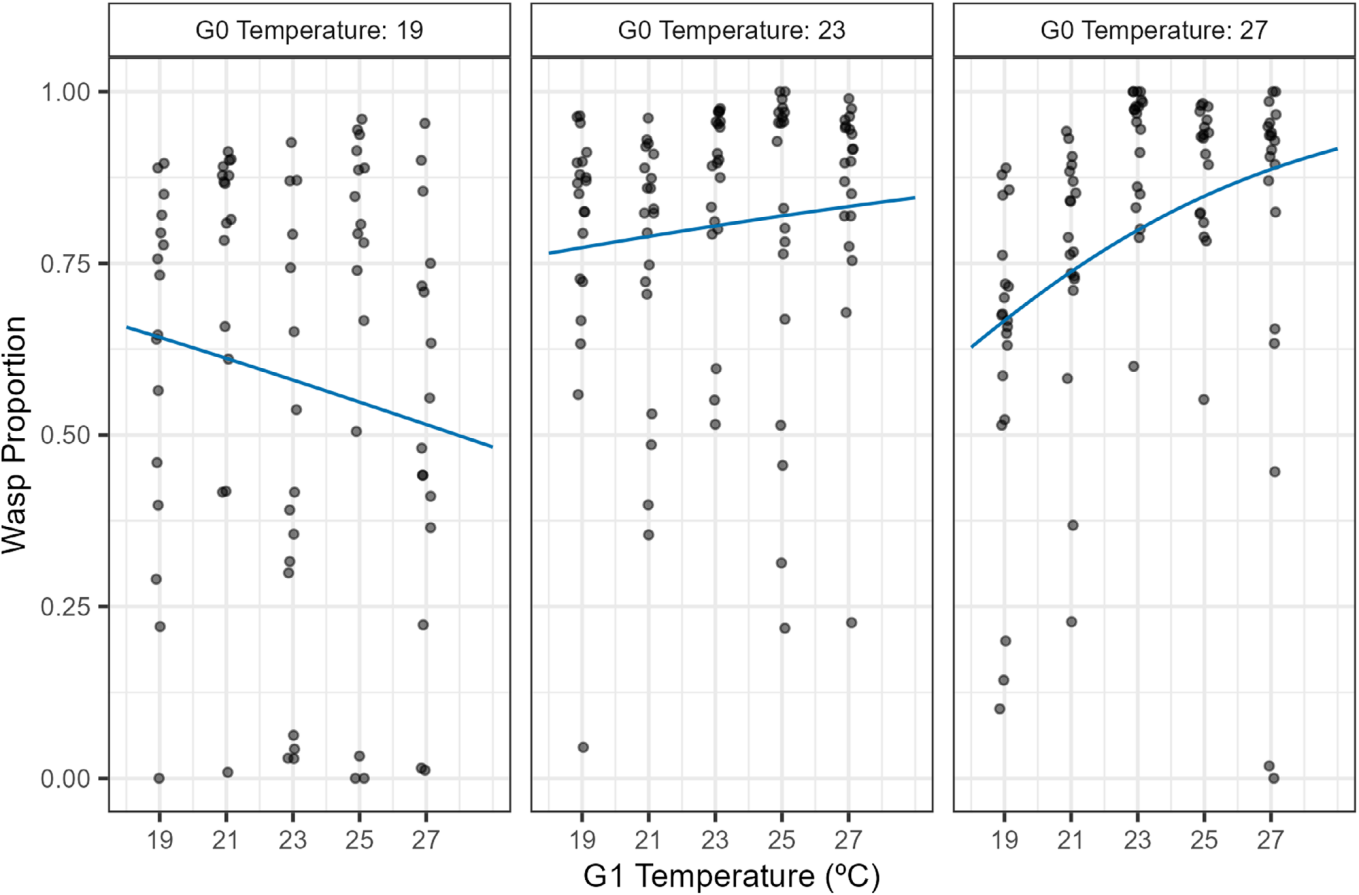
Wasp emergence rate best fit thermal performance curves and raw proportion data. Each panel shows the effect of temperature on the proportion of wasps (wasp count/total count, where total count includes both emerged flies and wasps) for each previous generation temperature (G0). See Figure 2 for delineation of generations. Black points represent raw data from each vial and are jittered horizontally. Means across the vials and approximations of standard errors for each treatment combination are given in Figure S4. Blue lines represent predictions based on best fit model. Fitted model parameter estimates and 95% confidence intervals are listed in Supporting Information (Table S2).

For the wasp temperature treatment experiment, the best supported model included G0 temperature as a categorical factor with three levels interacting with the effect of G1 temperature, indicating a significant transgenerational impact on wasp performance (Table 2, Model 2d). In comparison with the flies, the association between G1 temperature and wasp performance was more variable depending on G0 temperature (Figure 4). When G0 = 19°C, as G1 temperature increased, the predicted proportion of wasps decreased. In contrast, when G0 = 27°C, as G1 temperature increased, the predicted proportion of wasps increased. This could be interpreted as an adaptive transgenerational effect.

**TABLE 2:** Tests of the impact of G0 temperature on wasp performance through model comparison.

Model	Explanatory variable(s)	*n*	Likelihood	ΔAIC
2a	G1	2	−4913.71	1364.77
2b	G1 + G0	3	−4552.96	645.28
2c	G1*G0	4	−4397.26	335.87
2d	G1*factor (G0)	6	−4227.32	—

*Note*: The best model (2d) included distinct responses to the three previous generation temperatures and was much more strongly supported over models that included G0 temperature as a linear predictor. G0 = Temperature of wasp generation 0. G1 = Temperature of wasp generation 1.

### 3.2 Simulation model results

**FIGURE 5:**
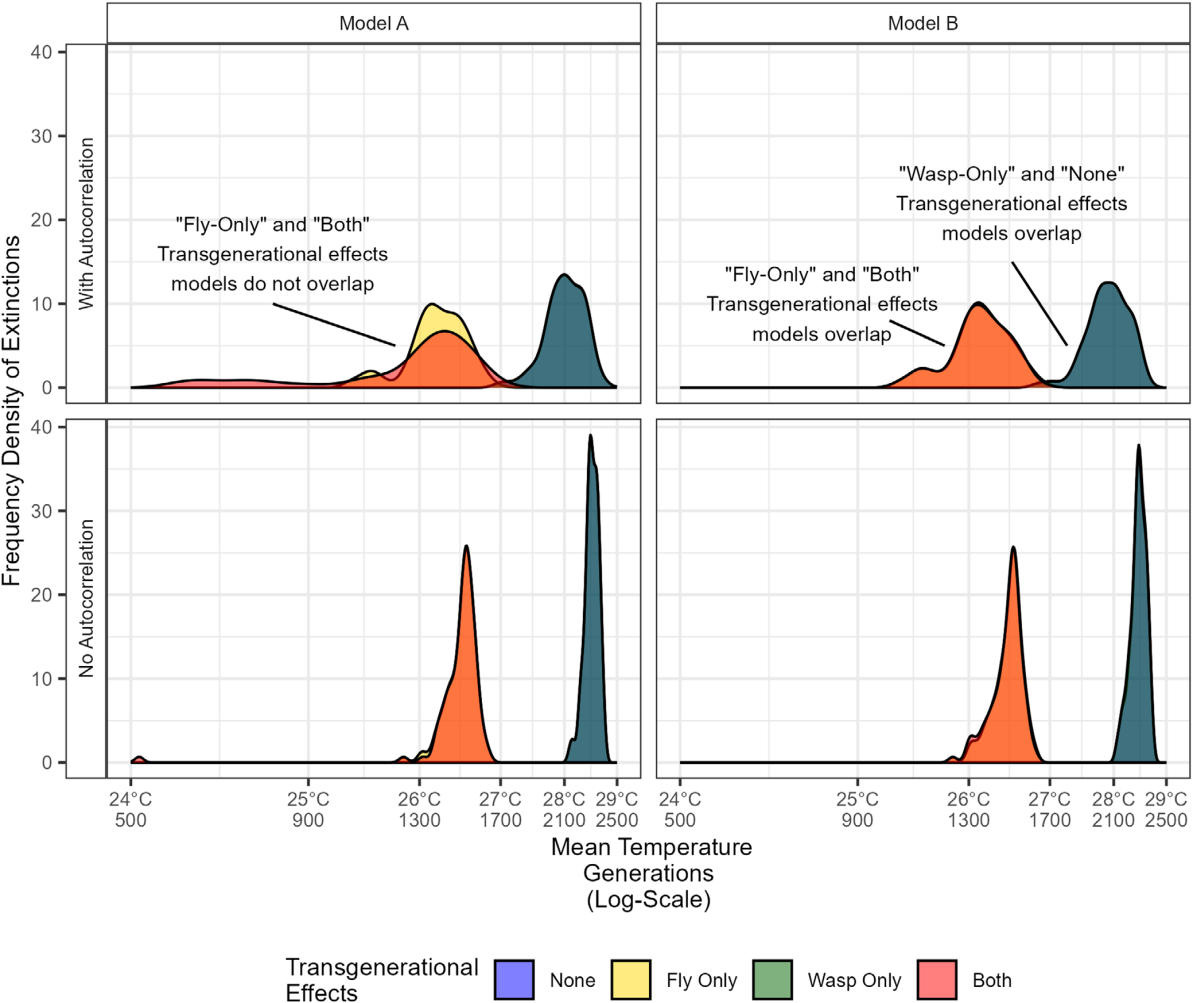
Identification of emergent dynamic impact of combined transgenerational effects. Density histograms of the observed distribution of extinction points over 100 simulations of the host–parasitoid system under different scenarios and models. The central temperature on which noise is imposed rises by 1°C per 400 generations after an initial 500 generation burn‐in period to allow the system to settle. Differences arise with joint inclusion of autocorrelation and both transgenerational effects. There is a near perfect overlap between scenarios with transgenerational effect modelled for ‘wasp‐only’ and ‘none’ (blue and green, combining to dark turquoise), as well as ‘both’ and ‘fly‐only’ (yellow and red, combining to orange), with the exception of the top left panel (simpler functional response and autocorrelation). Geometric means are given in Table S4. Note the logarithmic x‐scale.

Both autocorrelation and the full inclusion of transgenerational effects markedly hastened the simulated extinction of the fly host under both models (Figure 5, Table S4), reducing the mean temperature at which they could persist by nearly 2°C. In the simpler Model A, autocorrelation and the two transgenerational effects had a significant interactive effect (three‐way interaction term, *F*_1,693_ = 10.86, *p* = 0.001, Table S5). Taken separately, transgenerational effects on the fly had a markedly greater impact. Transgenerational effects on the wasp alone had minimal impact, but in combination with transgenerational effects on the fly and autocorrelation, further hastened extinction (Figure 5, top left). However, under Model B, there were no detectable interactive effects between either of the transgenerational effects and autocorrelation on the log‐time‐to‐extinction (*F*_1,693_ = 0.0083, *p* = 0.927, Table S5).

## 4 DISCUSSION

These results demonstrate how transgenerational effects can have substantial impacts on the consequences of climate change through a variety of pathways. Despite hope and interest in the potentially resilience‐enhancing nature of adaptive responses (Sohlström et al., 2021), our results highlight the negative aspects of transgenerational effects. Modelling is crucial to contextualise the various processes (Terblanche & Hoffmann, 2020), but our observation of only seeing emergent dynamic consequences of transgenerational effects with certain model forms emphasises the need for caution and further theoretical developments.

Two key caveats need to be reiterated when interpreting these results: Firstly, our focus was on identifying potential impacts of cross‐generational transfer of population dynamics rather than isolating physiological mechanisms. As such, although the nature of these responses suggests contributions from both condition‐transfer effects and potential adaptive transgenerational plasticity, these cannot be cleanly differentiated within our design. This is a fundamental challenge—while the observed effects are ‘transgenerational’ from the perspective of standard population models that track adults (Hassell, 2000), it is plausible that the most important underlying physiological processes can be considered within‐generational (Donelson et al., 2018) from other perspectives or framings (e.g. egg‐to‐egg). Secondly, these models are not intended to be specific representations of particular real‐world scenarios or locations. The experiments focus on just two species from a complex community (Chen & Lewis, 2023, 2024; Jeffs et al., 2021) and do not account for many features that would affect climate change responses within a fluctuating environment, such as microclimates (Pincebourde & Woods, 2020), competition (Terry, 2025), the impact of seasonality (Tougeron et al., 2020) and the potential for evolution (Chevin & Bridle, 2025; Rudman et al., 2022).

### 4.1 Experimental observations

We found clear experimental evidence that the parental thermal environment had a significant and substantial impact on subsequent reproductive output in both the hosts and parasitoids. The somewhat discordant responses of the host reproductive output and the parasitism rates to temperatures across generations open up the potential for complex dynamics to emerge.

When the parental fly generation was maintained in a thermally stressful environment (G0 = 27°C), subsequent reproductive output was lower across the temperature range (Figure 3), in line with previous results using *D. melanogaster* that identified elevated costs in extreme thermal environments, with high and constant temperatures reducing the thermal performance of the offspring generation (Cavieres et al., 2019). The most direct explanation is that G0 flies that developed in stressful temperatures were not able to take advantage of more clement conditions in the G1 generation. On average, across all offspring generation temperatures, the best fly performance was when the parental generation was maintained at an intermediate temperature (G0 = 23°C, Figure 3). This indicates a condition‐transfer effect and aligns with the optimum parental temperature hypothesis that parents kept at intermediate temperatures produce fitter offspring compared to parents at temperature extremes (Cohet & David, 1978; Gilchrist & Huey, 2001). Although our experimental design cannot distinguish developmental success rates from the number of eggs laid, it does reiterate the importance of accounting for potential carry over effects when assessing thermal performance (Jenkins & Hoffmann, 1994; Schiffer et al., 2013; Sgrò & Hoffmann, 1998).

There was also some indication of adaptive cross‐generational effects where offspring facing the same environmental conditions as their parents have a higher fitness than offspring facing different environmental conditions (Gilchrist & Huey, 2001) in anticipation of what they are likely to experience (Cavieres et al., 2020; Tariel et al., 2020). For instance, when the parental generation was maintained in a thermally stressful environment (G0 = 27°C), the proportional decrease in reproduction was lower when the offspring generation was also in a thermally stressful environment (Figure 3). This could indicate the possibility of some anticipatory effect, although it is overwhelmed by the non‐adaptive transfer of poor condition. Furthermore, the greatest thermal performance at 19°C was when the parental generation was also maintained at 19°C (Figure 3). This highlights that while it is important to differentiate between anticipatory and condition‐transfer effects when understanding the evolutionary causes and consequences of transgenerational effects (Sánchez‐Tójar et al., 2020), both can operate simultaneously.

The assay we used (with a discrete window of egg laying time) is especially sensitive to the rate of egg laying but does not capture the longevity of the G0 flies. While fly reproduction was lower when the parental generation was maintained in a thermally stressful environment, demographic trade‐offs may have meant other traits, such as thermal tolerance (Bozinovic et al., 2011) or longevity (Ismaeil et al., 2013), were not. In a previous study, *D. melanogaster* subjected to a variable and stressful thermal environment exhibited reduced reproduction, yet showed increased thermal tolerance compared to flies kept at a constant temperature (Cavieres et al., 2020).

The wasp thermal performance curves show clear evidence for improvements in thermal performance where there was a close temperature match between the generations. When the parental generation was maintained at a low temperature (G0 = 19°C), the greatest wasp proportion was when the G1 temperature was also low (Figure 4). In comparison, when the parental generation was maintained at a high temperature (G0 = 27°C), the greatest wasp proportion was when the temperature was also high. This acclimation would be expected to reduce their vulnerability to climate change despite many of their life‐history traits, such as a high trophic position and typical trophic specialisation, increasing their susceptibility (Jeffs & Lewis, 2013). We cannot, however, concretely identify if these changes are due to changes in host resistance, wasp virulence or changes to the functional response with our experimental design. We also note that in our experimental design using standardised host vials, we were not able to test if there was an interactive effect between the parental temperature of the host and their susceptibility to the wasp.

### 4.2 Time‐to‐extinction: Role of transgenerational effects and autocorrelation in temperature trajectories

The simulation models predict that including transgenerational effects of thermal conditions significantly hastens the time‐to‐extinction of this host‐parasitoid system. The substantial negative effect of including the transgenerational effects in the simulation models highlights the predominance of negative condition‐transfer over any potential adaptive transgenerational plasticity through anticipatory effects. Autocorrelation, even without transgenerational effects, magnifies the threat posed by environmental stochasticity (Lande et al., 2003). By prolonging periods of adverse conditions and reducing the presence of temporal refugia, positive temporal autocorrelation can amplify environmental stress and exacerbate the risk of extinction (Postuma et al., 2020).

Without autocorrelation, there should be no special dynamic consequences of transgenerational effects. The observed strong impact seen without autocorrelation shows that effectively averaging across assays of the thermal performance of flies is inflating the estimated performance at higher temperatures. Only the additional effects from the combination of autocorrelation and transgenerational effects are the direct dynamic impact from the transgenerational effects. In the simpler Model A, there was a clearly identifiable additional interaction effect, although this dynamic component was considerably smaller than the independent impacts of both autocorrelation and the direct effects of transgenerational effects. The modelled transgenerational effects observed in the wasps had essentially no impact on the time of fly extinction on their own, but under Model A did have an impact in combination with the transgenerational effects observed in the flies. The double effect of sequential host generations of reduced host and improved wasp performance brought forward the point at which the host population was pushed below the extinction threshold. This negative effect highlights how any benefits of acclimation were overwhelmed by condition‐transfer effects. By contrast, no interactive effect is detected within Model B. Both models are relatively standard representations of host–parasitoid interactions, while Model B assumed spatial clumping through the negative binomial term, which might act as one of the buffering mechanisms that absorb the emergent effect. The distinct responses under the two models highlight the context dependency of whether experimentally identified effects will have population‐level consequences.

### 4.3 Linking experiments, physiological mechanisms and dynamic consequences

Precisely distinguishing the multiple possible contributing factors that can underlie acclimatory and transgenerational effects is a significant challenge (Terblanche & Hoffmann, 2020), further confounded by difficulties precisely delimiting the boundaries between generations (Burggren, 2015; Lee et al., 2020) and the potential for influences to span multiple generations (Tariel et al., 2020). For example, environmental impacts on primordial germ cells that appear as examples of transgenerational plasticity could be due to within‐generation plasticity in offspring during early stages of development (Donelson et al., 2018).

Our broad‐scale approach allows us to deploy the high replication necessary to confidently identify effects but cannot differentiate physiological mechanisms, and the experimental requirements to fully partition all potential different processes can quickly become unfeasible at scale. While further experiments could address some of the gaps, for example, transferring a controlled number of eggs to differentiate the impact of adult condition influencing egg laying rate from effects related to offspring development at different temperatures, it is hard to assess how much difference such extra information would bring. As such, our results should be taken as experimentally informed potential scenarios, rather than precise forecasts. In scenarios with stochastic environments and multiple interacting species, model predicted outcomes are readily qualitatively changed under different parameter combinations (Terry et al., 2022). Closing the complexity gap and determining the appropriate level of abstraction to tackle these processes at scale will be a key challenge for future research. Our experiments were designed to align with models of host–parasitoid dynamics that conventionally operate on an adult–adult basis (Nicholson & Bailey, 1935), but demarking the generational boundary at a different stage (e.g. eggs–eggs) could well give different results. Temperature affects insect life stages differently (Pawar et al., 2024), so our generational‐scale treatment may miss key impacts by ignoring demographic structure (Amarasekare & Coutinho, 2013).

## 5 CONCLUSION

Our results empirically highlight the substantial, but context‐dependent, impact multi‐trophic transgenerational effects can have on population dynamics under climate change. These findings underscore the need to quantify and contextualise transgenerational effects when understanding species' responses to climate change and show the need to move beyond demonstrations that such effects exist towards improving our theoretical understanding of the scenarios where they are influential.

## AUTHOR CONTRIBUTIONS

Natalie L. Bright conducted the experiments and wrote the first manuscript draft. J. Christopher D. Terry supervised the project, designed the analyses and finalised the manuscript. Jinlin Chen co‐designed the experiment and contributed significantly to manuscript development. All authors gave final approval for publication.

## CONFLICT OF INTEREST STATEMENT

The authors declare no conflict of interest.

## STATEMENT ON INCLUSION

Our study is a coupled laboratory and theoretical study conducted in the United Kingdom and part of a wider global research programme on this insect community.

## Supporting information


**Table S1.** Parameter estimates and their 95% confidence intervals for the fly thermal performance curves.
**Table S2.** Parameter estimates and their 95% confidence intervals for the wasp thermal performance curves.
**Table S3.** Description and values of parameters used in the simulation models.
**Table S4.** Mean time to extinction, in generations, of the host‐parasitoid system depending on the inclusion of temporal autocorrelation in temperature and transgenerational effects of thermal conditions in the simulation model.
**Table S5.** Supporting statistics for difference between simulation treatments under each model.
**Figure S1.** Deriving fly population growth rate.
**Figure S2.** Example time series from simulations.
**Figure S3.** Standard error around the mean for each temperature treatment combination.
**Figure S4.** Depiction of standard error around the mean proportion across the replicate vials for each temperature treatment combination.

## Data Availability

Raw data, R code, fitted model objects and R Markdown documents covering all steps are publicly archived on Zenodo https://doi.org/10.5281/zenodo.13327763 (Terry et al., 2025) and available through GitHub at https://github.com/jcdterry/MultiGenHeatEffects_public.
